# Development and Challenges of Pre-Heart Failure with Preserved Ejection Fraction

**DOI:** 10.31083/j.rcm2409274

**Published:** 2023-09-25

**Authors:** Guoju Dong

**Affiliations:** ^1^Department of Cardiovascular Internal Medicine, Xiyuan Hospital, Chinese Academy of Traditional Chinese Medicine, 100091 Beijing, China; ^2^National Clinical Research Center for Chinese Medicine Cardiology, Xiyuan Hospital, Chinese Academy of Traditional Chinese Medicine, 100091 Beijing, China

**Keywords:** pre-heart failure (pre-HF), heart failure with preserved ejection fraction (HFpEF), asymptomatic heart failure

## Abstract

Pre-heart failure with preserved ejection fraction (Pre-HFpEF) is a critical link to the development of heart failure with preserved ejection fraction (HFpEF). Early recognition and early intervention of pre-HFpEF will halt the progression of HFpEF. This article addresses the concept proposal, development, and evolution of pre-HFpEF, the mechanisms and risks of pre-HFpEF, the screening methods to recognize pre-HFpEF, and the treatment of pre-HFpEF. Despite the challenges, we believe more focus on the topic will resolve more problems.

## 1. Introduction

Pre-heart failure (pre-HF) was first formally proposed in the 2022 American Heart Association (AHA)/American College of Cardiology (ACC)/Heart Failure Society of America (HFSA) guidelines for the management of HF [1], a new terminology to replace stage B, first defined in the 2001 ACC/AHA guidelines for the evaluation and management of chronic heart failure in adults [2]. Pre-HF and stage B of HF refers to left ventricular (LV) dysfunction without developed symptoms. The concept of pre-HF is to emphasize the progressive nature of HF and remind physicians to prevent HF as early as possible. LV systolic dysfunction (LVSD) and LV diastolic dysfunction (LVDD) are both symptoms of pre-HF. However, the emphasis is that all stage B guidelines mostly relate to individuals with asymptomatic LVSD due to a lack of awareness of HF with LVDD [3].

**Fig. 1.**
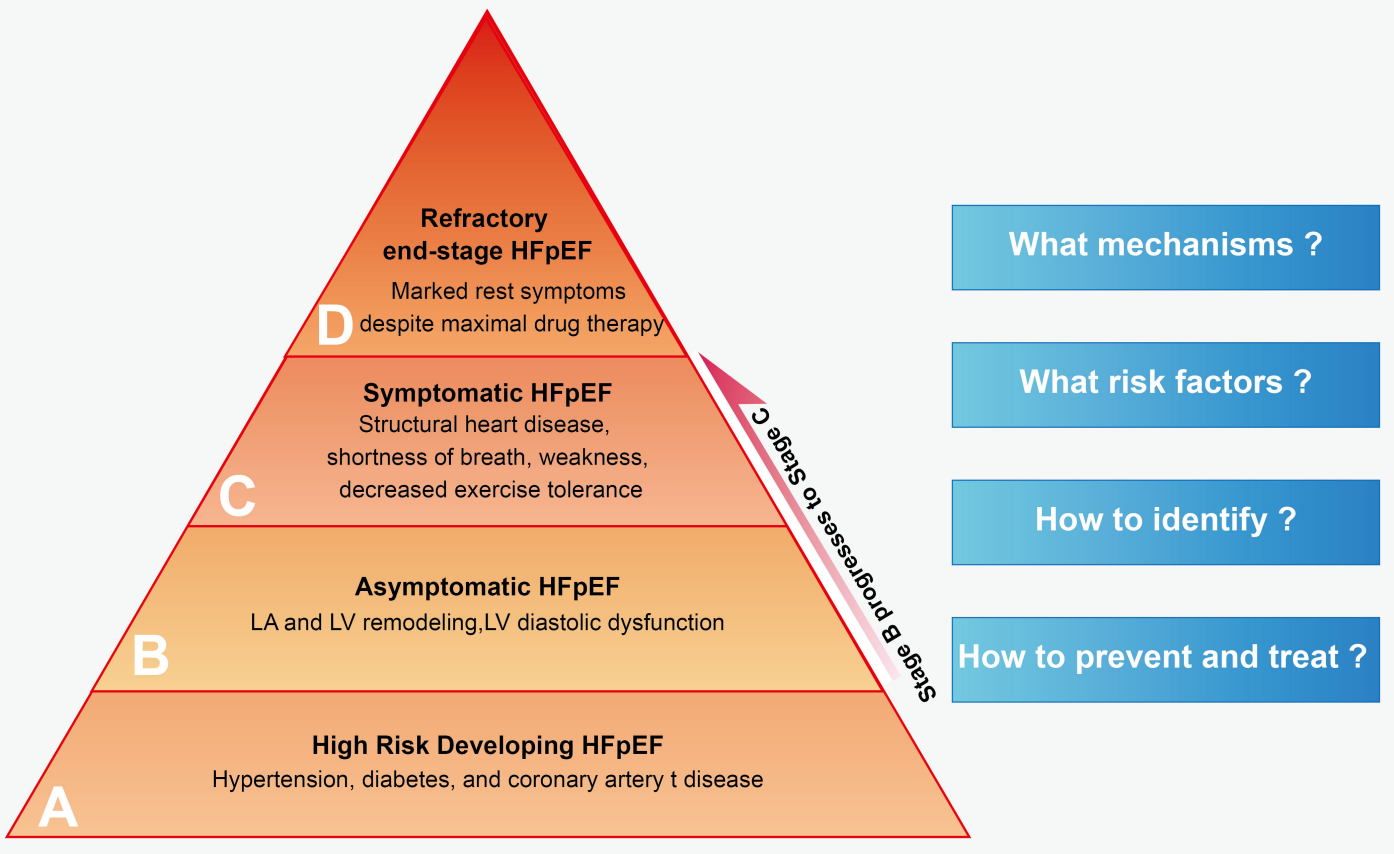
**Staging of HFpEF**. HFpEF, heart failure with preserved ejection fraction; LA, left atrial; LV, left ventricle.

HF with preserved ejection fraction (HFpEF), formerly known as diastolic HF, was first created to describe the patients who had HF but not with a major reduction in systolic function in the 2012 European Society of Cardiology (ESC) guidelines for the diagnosing and treating acute and chronic heart failure [4]. HFpEF was formally proposed in the 2016 ESC HF guidelines to highlight the symptoms of HF patients with normal LV ejection fraction (LVEF), who generally do not have a dilated LV, but instead often have increased LV wall thickness and/or increased left atrial (LA) size as a sign of increased filling pressures [5]. With active primary prevention efforts, the incidence of heart failure with reduced ejection fraction (HFrEF) has decreased in recent years [6]. However, the incidence of HFpEF has surged dramatically [7, 8], especially in the past decade, and is closely associated with growing comorbidities such as obesity and metabolic syndrome epidemics [9, 10, 11, 12]. Although HFpEF affects half of all patients with HF worldwide, few treatments have proven effective, making it the most severe medical need in cardiovascular disease and continues to be a difficult challenge for clinicians [13]. To better understand and control HFpEF, we need to focus on pre-HFpEF. Pre-HFpEF lacks signs or symptoms of HF, but has preserved LVEF with incipient structural changes similar to HFpEF, and possesses elevated biomarkers of cardiac dysfunction [14]. There has been some progress in the transition from asymptomatic pre-HFpEF to symptomatic HFpEF but some challenges as well (Fig. 1).

## 2. Mechanisms of Pre-HFpEF

HFpEF develops from pre-HFpEF, while pre-HFpEF is caused by LV dysfunction. Even if LV dysfunction comprises LVSD and LVDD, LVDD is the leading factor of LV dysfunction. The most conspicuous and unifying hemodynamic alteration in pre-HFpEF is an elevation in LV filling pressures caused predominantly by LVDD [15]. Diastolic heart function includes LV relaxation and compliance. The former refers to the change in intracavitary pressure per unit of time, and the latter is that per unit volume. LV compliance impairment, rather than LV relaxation abnormality, plays a crucial role in the induction of pre-HFpEF [16, 17], which results in an elevation in filling pressures, further increases Left ventricular end-diastolic pressure (LVEDP) and LA pressure [18]. If the process cannot be effectively prevented in time, it will develop into symptomatic HF (stage C).

## 3. Risk Factors of Pre-HFpEF

**Fig. 2.**
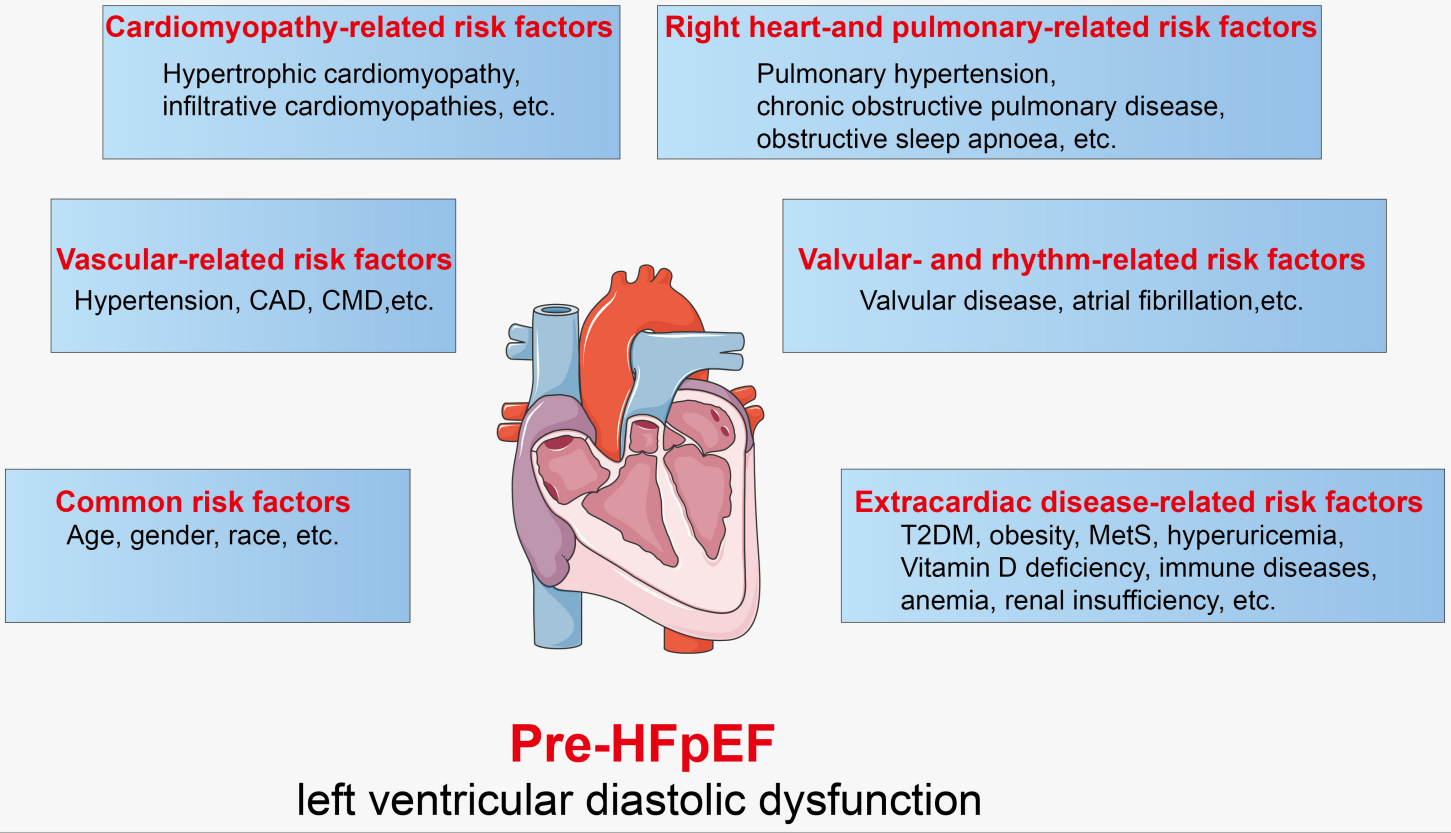
**Risk factors of pre-HFpEF**. CAD, coronary artery disease; CMD, coronary microvascular dysfunction; T2DM, type 2 diabetes mellitus; MetS, metabolic syndrome; HFpEF, heart failure with preserved ejection fraction.

The pathological mechanisms of pre-HFpEF remind us to determine the risk factors impairing ventricular compliance. Unfortunately, many alterable and unalterable risk factors are involved, making HFpEF a challenging heterogeneous syndrome. Since no unified treatment is available for diverse-cause HFpEF, it is vital to discover the cause and prevent it from developing. Ge J. [19] proposed aetiology-oriented phenotype and classification, which is practical and beneficial to the clinic. With the increasing emphasis on pre-HFpEF, more risk factors and aetiology were supplemented on Ge’s proposal (Fig. 2).

### 3.1 Common and Unalterable Risk Factors

Age is one of the primary risk factors associated with HFpEF. The majority of older adults in the community are at risk of HF (stages A or B), and at least two-thirds of older adults with prevalent HF (stage C) are HFpEF [20, 21], meaning that pre-HFpEF is more likely to occur in older populations. Women are more prone to develop HFpEF than men [21, 22]. The underlying pathophysiological mechanisms may include hormonal differences and bio-hormonal system activity associated with various cardiovascular risk factors [23]. Racial and ethnic disparities also influence the incidence of pre-HFpEF. Non-Hispanic Black beneficiaries had a slightly lower incidence of HF than non-Hispanic White beneficiaries from 2011 to 2016 [24]. While Asian/Pacific Islander patients had a similar incidence of HF to non-Hispanic White patients, but a lower rate of death [25].

### 3.2 Vascular-Related Risk Factors

Hypertension increases cardiac afterload and gradually induces cardiac remodelling, consisting of LVDD and concentric LV hypertrophy [26]. Subclinical changes to LV strain and diastolic function can be found before the development of decreased LVEF even among young people with hypertension [27]. Moreover, coronary artery disease (CAD) and coronary microvascular dysfunction (CMD) are major contributors to the pathophysiology of HFpEF. However, CMD is often neglected in clinics because microvessels are invisible by the current imaging techniques [28]. The PROMIS-HFpEF research revealed that in the absence of unrevascularized macrovascular CAD, CMD was more prevalent in HFpEF patients [29]. Meanwhile, a cohort study found that 91% of patients with HFpEF had epicardial CAD, CMD, or both [30].

### 3.3 Cardiomyopathy-Related Risk Factors

Cardiomyopathies, an increasingly important cause of HFpEF, are a heterogeneous group of heart muscle diseases [31]. Cardiomyopathies, such as hypertrophic cardiomyopathy [32], restrictive cardiomyopathies [31], infiltrative cardiomyopathies like cardiac amyloidosis [33], fabry cardiomyopathy [34], transthyretin amyloid cardiomyopathy [35], and tumour-related cardiac toxicity [36], can contribute to impaired cardiac compliance.

### 3.4 Right Heart- and Pulmonary-Related Risk Factors

Pulmonary hypertension is the most common cause of pre-HFpEF [37]. Right ventricular dysfunction (RVD) is found in 4%–50% of patients with HFpEF. Although RVD is often complicated by PH, the development of RVD in HFpEF may also be induced by other comorbidities, such as chronic obstructive pulmonary disease, obstructive sleep apnoea, and atrial fibrillation (AF) [38], each of which can also cause pre-HFpEF.

### 3.5 Valvular- and Rhythm-Related Risk Factors

With global aging, the incidence of degenerative heart valve disease has increased dramatically, which is a crucial factor in pre-HFpEF. Although many heart valve disease patients claimed to be asymptomatic, exercise testing revealed the objective occurrence of symptoms, indicating the existence of pre-HFpEF [39, 40]. AF shares similar risk factors and many common clinical features with HFpEF [41, 42], and sometimes, it is hard to differentiate whether HFpEF or AF occurs first, and there is a very close and intricate relationship between them, making them seem like vicious twins [43]. In the Framingham Heart Study, AF was identified as a major risk factor for new-onset HFpEF with a hazard ratio (HR) of 2.5, and the presence of AF was more predictive of incident HFpEF than HFrEF with a HR of 2.3 [44]. Despite the tight relationship between pre-HFpEF and AF prevalence, it might be arduous to diagnose pre-HFpEF when AF is present because of the overlap of comparable clinical features [45].

### 3.6 Extracardiac Disease-Related Risk Factors

#### 3.6.1 Metabolic Diseases

3.6.1.1 Type 2 diabetes mellitus

Almost half of asymptomatic type 2 diabetes mellitus (T2DM) patients have LVDD, and more than a third of them exhibit a moderate LVDD pattern with increased B-type natriuretic peptide (BNP), suggesting a significantly increased risk of HFpEF [46]. Compared with pre-HF patients without T2DM, those with T2DM were more likely to have HF [47].

3.6.1.2 Overweight and obesity

Being overweight or obese could increase afterload on the heart, and these individuals are at increased risk of HF. Mild obesity can lead to cardiac structural changes, including LV hypertrophy and LV enlargement, and severe obesity can result in LVDD and LVSD [48]. Of course, obesity or being overweight seldom exist alone and will further deteriorate LV dysfunction when accompanied with other risk factors [49].

3.6.1.3 Metabolic syndrome

Metabolic syndrome represents a cluster of interrelated common clinical disorders, and is associated with a high prevalence of LVSD and LVDD [50]. Research shows excessive visceral fat accompanied by adipocyte dysfunction is an independent determinant of LA volume, E/A, and E/$e^{\prime }$, which may be a more significant factor than glycemic control in developing LVDD and LV hypertrophy in T2DM [51].

3.6.1.4 Hyperuricemia

Hyperuricemia is associated with unfavourable cardiac remodeling and is closely related to LVDD even at a relatively low clinical cut-off. LVDD can be aggravated among patients with gout, especially for women with hyperuricemia or gout [52].

#### 3.6.2 Vitamin D Deficiency

Vitamin D deficiency is prevalent in HF [53, 54]. 25-hydroxyvitamin D [25(OH)D], which is the essential circulating vitamin D metabolite and a good indicator of vitamin D status [53, 55], is relevant to the early stages of HFpEF. Lower 25(OH)D levels are significantly and independently associated with reduced functional capacity in patients with LVDD or newly diagnosed HFpEF [56].

#### 3.6.3 Immune Diseases

Immune diseases, including rheumatoid arthritis and systemic lupus erythematosus [57], can affect the cardiac vasculature, valves, myocardium, pericardium, and conduction system, leading to a plethora of cardiovascular manifestations, which may be mild and clinically silent or can increase substantial cardiovascular morbidity [58]. Rheumatoid arthritis features systemic inflammation [59] and carries a two fold increased incidence of HFpEF [60]. Before that, it is often related to LVDD during asymptomatic pre-HFpEF [61].

#### 3.6.4 Hyperdynamic Circulatory State

Anaemia leads to inadequate blood supply and cardiac overload, which is more prevalent in HFpEF than in HFmrEF and HFrEF [62]. Liver diseases, such as nonalcoholic fatty liver disease [63] and advanced fibrosis, are independently associated with incident HFpEF but not HFrEF [64], which suggests that risk factors or mechanisms for liver disease may have multiple overlap syndromes with those with HFpEF rather than HFrEF. Moreover, hyperthyroidism, a condition similar to anaemia, can lead to increased cardiac oxygen uptake and LVDD, which is also an independent risk factor for pre-HFpEF [65].

#### 3.6.5 Other Diseases

Renal insufficiency is a common comorbidity in patients with HFpEF. An analysis of 118 patients with asymptomatic LVDD and 18 patients with HFpEF suggests that intrinsic renal insufficiency determines whether LVDD will become symptomatic [66]. Cardiac radiation exposure due to cancer radiotherapy contributes to coronary microvascular endothelial inflammation, and such disturbance is strongly associated with the development of HFpEF [67]. Over 60% of pre-eclampsia cases are present with concentric remodelLing and normal LVEF, and most pre-HFpEF will recover during follow-up, whereas approximately 20% of pre-HFpEF will develop over the next few years [68, 69]. In addition, a meta-analysis showed that chronic cocaine use might lead to abnormalities in cardiac structure and function, which are consistent with diastolic HF [70].

## 4. Identification of Pre-HFpEF

HF is a unidirectional irreversible process, and early identification of pre-HF would allow us to use cardioprotective medication and/or lifestyle modification to prevent or delay the progression to symptomatic HF [71, 72]. However, even for HFpEF, diagnosis is still a challenging problem, let alone pre-HFpEF [73]. The biggest bottleneck is that all patients with LVDD are at risk of HFpEF, so how should we recognize patients with HFpEF [74]? We need to weigh the benefits against the costs, neither over-screening nor under-diagnosis, and consider the accuracy and clinical feasibility of the diagnosis.

### 4.1 Echocardiography

Echocardiography is the primary method for evaluating LVDD [75]. LA remodelling and RVD are closely associated with increased LV filling pressures and LVDD [76]. Therefore, the assessment criteria of LVDD consists mainly of indicators reflecting LA structural or functional abnormality, LV relaxation and compliance, and RVD. Although some progress has been made, data and cut-off points of diagnostic value still need further research [77] (Table 1).

**Table 1.** **The cut-offs of the echocardiographic indices**.

Items	Index	Cut-offs
Indices related to LA remodelling	LAVI	≥29 mL/$m^{2}$
Indices related to LV remodelling	LVMI	>116 g/$m^{2}$ for man or >95 g/$m^{2}$ for woman
	Average E/eʹ	≥15
	Septal eʹ	<7 cm/s
	Lateral eʹ	<10 cm/s
	LVEF	>50%
	GLS	>–18%
Indices related to RV dysfunction	TRV	>2.8 m/s
	PASP	>35 mmHg

Abbreviations: LA, left atrial; LV, left ventricular; RV, right ventricular; LAVI, left atrial volume indices; LVMI, left ventricular mass index; E, mitral inflow peak early filling velocity; eʹ, early diastolic mitral annular velocity of the septal and lateral sites; LVEF, left ventricular ejection fraction; GLS, global longitudinal strain; TRV, Tricuspid regurgitation velocity; PASP, pulmonary artery systolic pressure.

#### 4.1.1 Indices Related to LA Remodelling

LA remodelling is independently associated with pre-HFpEF [78], including delayed LA contraction, shortened LA emptying, decreased LA compliance, and increased LA filling pressure [79]. Therefore, accurately assessing LA structure and function is the cornerstone in recognizing LVDD as a clinical precursor for (pre-)HFpEF [80]. LA dimension and LA maximum/minimum volume indices (LAVI) are popular criteria to evaluate LA size, which can predict the transition from pre-HFpEF to an overt symptomatic phase [81]. However, deterioration in LA function usually precedes structural changes [82, 83, 84]. LA reservoir strain (LARS) reflects the contemporaneous LA measure of diastolic function and is therefore a more sensitive LA marker of LVDD than LAVI [85, 86, 87]. Of course, adding LARS to LAVI would facilitate the identification of LVDD in patients with pre-HFpEF [88].

#### 4.1.2 Indices Related to LV Remodelling

LV hypertrophy, usually defined by current guidelines as an LV mass index (LVMI) >95 g/$m^{2}$ in women and >116 g/$m^{2}$ in men, is the most common cardiac complication of hypertension, which ultimately leads to pre- HFpEF [26]. The E/A ratio is the most conventional ultrasonic LVDD parameter, and the decrease in the E/A ratio is usually identified with the possibility of LVDD [21, 89]. However, the “normal” E/A ratio may be a pseudonormal or a supernormal mitral inflow pattern, which probably misleads the clinicians to make a wrong judgement. The E/$e^{\prime }$ ratio (mean of septal and lateral) is more sensitive to diagnose LVDD compared to the E/A ratio, which is associated with a similar degree of exercise capacity decline to LVSD and can distinguish B stage from A stage of HF [90, 91, 92]. Furthermore, HFpEF patients usually present with localized myocardial systolic dysfunction even if their LVEF remain normal [93, 94, 95]. Global longitudinal strain (GLS) can detect subtle changes in LV systolic function and independently predicts HF events in asymptomatic elderly patients with T2DM [96, 97], which has become a clinically feasible alternative to LVEF for measuring myocardial function [98, 99].

#### 4.1.3 Indices Related to RV Dysfunction

Tricuspid regurgitation velocity (TRV) reflects RVD. Pulmonary hypertension, which is defined as pulmonary artery systolic pressure (PASP) >35 mmHg and often calculated by TRV, is highly prevalent in HFpEF and can distinguish HFpEF from pre-clinical hypertensive heart disease without overt HF [100].

In summary, although echocardiography is validated, reproducible, and available, problems still exist. The cut-offs for different indices in different ages and races still need further confirmation [101, 102], and standardized technical operation is also necessary, especially in medically underserved areas [103]. In usual circumstances, to enhance diagnostic accuracy, a combination of multiple indicators is more reasonable than a single indicator [104]. In addition, to increase the detection of subclinical pre-HFpEF, stress/post-exercise echocardiographic testing would reveal LVDD more significantly [105].

### 4.2 Serum Biomarkers

#### 4.2.1 B-Type Natriuretic Peptide

B-type natriuretic peptide (BNP) or N-terminal prohormone-BNP (NT-proBNP) are used to establish a diagnosis or exclusion of HF, especially for symptomatic HF [106, 107]. However, it is debatable whether BNP or NT-proBNP is useful for pre-HFpEF [108]. BNP may help screen pre-clinical LV dysfunction at a cut-off of 50 pg/mL [109], but the cut-off of BNP exists as age-specific and race-specific [110]. A retrospective study showed NT-proBNP level alone is not associated with LVDD but with older age, females, lower BMI, and higher creatinine levels [111].

#### 4.2.2 Soluble Growth Stimulated Expression Gene 2 Protein

Soluble growth stimulated expression gene 2 protein (sST2) represents a member of the interleukin 1 receptor family [112]. A strong correlation exists between serum sST2 level and LVDD or LVEDP [113, 114]. The sST2 concentration was significantly lower in patients with E/$e^{\prime }$ <8 compared to those with E/$e^{\prime }$ 8–15 or E/$e^{\prime }$ >15 [115]. The multivariate analysis demonstrated that sST2 >13.5 ng/mL might be the threshold to predict pre-HFpEF. However, sST2 is still controversial in screening HFpEF. Some research did not find it was associated with LV structure, LVDD, or LVSD [116, 117].

#### 4.2.3 Galectin-3

Galectin-3 (Gal-3) is a β-galactoside-binding member of the lectin family, which is implicated in a chronic profibrotic process, and a promising novel biomarker for early detection of pre-HFpEF [118, 119], phenotyping [120], risk stratification [121, 122], and therapeutic targeting of HFpEF [123]. De Boer *et al*. [124] suggest that it is likely that Gal-3 may one day be included in the classification of “stage B HFpEF” and that, in particular, the combination of BNP and Gal-3 levels may detect patients with pre-HFpEF.

#### 4.2.4 Growth-Differentiation Factor-15

Growth differentiation factor-15 (GDF-15), a stress-responsive transforming growth factor-ß-related cytokine, is elevated in subjects with HFpEF and can differentiate normal LVDD from asymptomatic LVDD [125], indicating a possible novel biomarker for pre-HFpEF. However, GDF-15 is elevated similarly in both HFpEF and HFrEF, so is not a unique biomarker to distinguish between pre-HFpEF or HFpEF [126].

#### 4.2.5 IGF-1 and IGFBP-7

Insulin-like growth factor binding protein-7 (IGFBP-7) modulates the biological activities of insulin-like growth factor-1 (IGF-1), constituting the IGF1/IGFBP-7 axis and correlates with LVDD [127]. The plasma IGF1/IGFBP-7 concentrations ratio can readily distinguish patients with or without HFpEF. In other words, it is a pragmatic factor in differentiating pre-HFpEF [128].

#### 4.2.6 MMP9 and TIMP1

Matrix metalloproteinase 9 (MMP9) is mainly responsible for the dynamic balance of degradation and remodelling of the extracellular matrix. Tissue inhibitors of metalloproteinase 1 (TIMP1) could degrade MMP9 and reduce collagen degradation at a tissue level. The elevated MMP9/TIMP1 ratio, in particular, is associated with LA remodelling and reduced chamber compliance [129], which raises the possibility of earlier detection of pre-HFpEF at risk of evolution to HF and may help develop effective preventative strategies [130].

#### 4.2.7 Endostatin

Endostatin is a circulating endogenous angiogenesis inhibitor and a potential new HF biomarker [131]. Endostatin serum levels are significantly elevated in patients with asymptomatic LVDD, acting as indicators of pre-HFpEF, and correlate with NT-proBNP [132].

#### 4.2.8 Urinary Peptidomics Biomarker

Urine contains an array of low-molecular-weight peptides, approximately 60% comprising collagen fragments [133]. Since pre-HFpEF is characterized by extracellular matrix alterations and particular collagen homeostasis, urine is a more suitable and stable biological source than blood when identifying collagen peptides and predicting LVDD [134]. The urinary proteome is well-characterized, and reference standards are available [135]. Urinary peptidomic biomarkers, which consist of a set of urinary peptides specific for LVDD, constitute a high-dimensional model (classifier) and serve as a sensitive tool to forecast pre-HFpEF and improve the risk stratification of HFpEF [136, 137, 138, 139].

### 4.3 Cardiovascular Magnetic Resonance

Cardiovascular magnetic resonance can predict elevated LV filling pressure, which is non-inferior to right heart catheterization-measured pulmonary artery wedge pressure, and significantly improves the classification provided by standard echocardiography assessment for suspected HF patients [140]. A fully-automatic deep learning method for myocardial strain analysis based on Cardiovascular magnetic resonance cine images can detect pre-HFpEF in young adults with cardiac risk factors [141].

### 4.4 Composite Index Prediction Models

At present, no single gold index can confirm pre-HFpEF, while combining different indicators can improve diagnostic accuracy. Therefore, composite index prediction models emerge when necessary. Considering different variables and numerous biomarkers, machine-learning-derived models or artificial intelligence-based analyses are necessary for analyzing and predicting pre-HFpEF [142, 143, 144]. Besides combining different biomarkers, we can also combine cardiac ultrasound indicators with biological indicators to increase the predictive effect of pre-HFpEF [145]. LV hypertrophy (LV septum >11 mm) and elevated biomarkers (NT-proBNP >40 pg/mL) or troponin (T >0.6 pg/mL) can demonstrate high LV myocardial stiffness compared to age- and sex-matched healthy controls, which appears to represent a transitional state from a normal healthy heart to HFpEF and could be a key marker of future HFpEF [146]. In addition, the HFA-PEFF score was developed to aid in the early identification of HFpEF [147]. The $H_{2}$FPEF also helps discriminate the likelihood of HFpEF with a total score of ≥6 points [73, 148], While a high percentage of patients with an $H_{2}$FPEF score of 2–5 points shows a strong association with LVDD, which suggests it is applicable in predicting future HFpEF, especially when combining stress tests [149, 150].

## 5. Treatments of Pre-HFpEF

Although multiple studies highlight the benefit of pharmacotherapy for pre-HFrEF, studies of specific treatments to alter the onset of HF in asymptomatic cardiac dysfunction with HFpEF are limited [1]. The main reason is the high heterogeneity in the aetiology of pre-HFpEF. Thus aetiology-specific treatment options are vital preventions in the primary transition from stage A to pre-HFpEF. It is important to actively interfere during stage A (patients at risk for HFpEF), including maintaining healthy lifestyle habits (such as regular physical activity, normal weight, and healthy dietary patterns), controlling blood pressure, and regulating glucolipid metabolic balance. Nevertheless, causative therapies are not available, especially regarding cardiomyopathies, immune diseases, and tumour-related cardiac toxicity [151].

Rich clinical evidence of Angiotensin Receptor-Neprilysin Inhibitor and Sodium-glucose co-transporter protein inhibitors has been discovered for treating HFpEF [152, 153, 154], but further research on their effects in pre-HFpEF needs to be done. Nevertheless, it is still worth looking forward to new treatments. Up to now, exercise training may be the only proven effective treatment for pre-HFpEF. Exercise training can reduce LV myocardial stiffness and may protect against the future risk of HFpEF, but it may mainly be limited in middle-aged patients with pre-HFpEF [155]. As it is hard to reverse cardiac atrophy and stiffening for elderly patients, it is necessary to limit the amount of exercise in these individuals [156, 157].

## 6. Challenges and Perspectives

HFpEF is a heterogeneous clinical syndrome associated with multiple risk factors, including traditional cardiovascular and non-cardiovascular risk factors. HFpEF is also a progressive disease in which patients transition from asymptomatic pre-HFpEF to symptomatic HFpEF. Therefore, early recognition and cardioprotective therapies in asymptomatic pre-HF are crucial and require close multidisciplinary cooperation. Fortunately, we have made some progress on the recognition of pre-HFpEF. Nevertheless, there are still numerous challenges to resolve. First of all, the definition of pre-HFpEF has already reached a consensus in AHA/ACC/HFSA guidelines. However, the cut-offs for various indices for patients of different ages and ethnicities still need to be confirmed. Secondly, clinicians should routinely assess patients’ risks for potential pre-HFpEF symptoms and instruct patients on common risks such as hypertension, coronary artery disease, and diabetes. Finally, it is vital to establish a screening method for pre-HFpEF considering the cost-effectiveness of different strategies, technical feasibility and repeatability in various countries and regions. It is a fact that standardized screening for pre-HFpEF remains challenging among populations due to the heterogeneity of risk factors [158]. Worse still, common indicators have limitations. For example, BNP shows poor specificity and wide biological variability and can hardly ever be used alone for screening pre-HFpEF. Echocardiography requires technical standardization and stability. Certain biomarkers are too costly and unavailable.

Despite the scientific and clinical challenges, progress in the understanding of pre-HFpEF might promote diagnostic and therapeutic modalities [159]. Firstly, pre-HFpEF involves multiple disciplines and needs interdisciplinary cooperation. A better understanding of the physiologic phenotypes of HFpEF patients may allow for better and more tailored treatment and prognosis prediction in pre-HFpEF patients. Moreover, since not all the patients of pre-HFpEF will develop symptomatic HFpEF, it is necessary to carry out risk stratification according to the screening results or establish prediction models to identify higher-risk patients who need a long-term follow-up in the clinic. Finally, although no treatment has yet been shown to reduce the morbidity of symptomatic HFpEF, we still expect the emergence of evidence for new treatment methods and measures, especially in traditional Chinese medicine which focuses on the idea that “a Saint does not treat the disease but prevents the disease” and is good at multi-target intervention.

## 7. Conclusions

In conclusion, HFpEF is a heterogeneous syndrome with multiple risk factors. The concept of pre-HFpEF was developed to emphasize the progressive nature of HF and remind physicians to prevent HF as early as possible. Therefore, early recognition and cardioprotective therapies in asymptomatic pre-HFpEF are crucial. A focus on pre-HFpEF and further research is vital to improve the treatment and reduce the harm caused by HFpEF to patients.

## Abbreviations

HF, heart failure; LV, left ventricular; HFpEF, heart failure with preserved ejection fraction; LVEF, left ventricular ejection fraction; LA, left atrial; HFrEF, heart failure with reduced ejection fraction; LVEDP, left ventricular end-diastolic pressure; CAD, coronary artery disease; CMD, coronary microvascular dysfunction; RVD, right ventricular dysfunction; AF, atrial fibrillation; HR, hazard ratio; T2DM, type 2 diabetes mellitus; LAVI, left atrial volume indices; LARS, left atrial reservoir strain; LVMI, left ventricular mass index; GLS, global longitudinal strain; TRV, Tricuspid regurgitation velocity; PASP, pulmonary artery systolic pressure; BNP, B-type natriuretic peptide; NT-proBNP, N-terminal prohormone-B-type natriuretic peptide; sST2, soluble growth stimulated expression gene 2 protein; Gal-3, galectin-3; GDF-15, growth differentiation factor-15; IGFBP-7, insulin-like growth factor binding protein-7; IGF-1, insulin-like growth factor-1; MMP9, matrix metalloproteinase 9; TIMP1, tissue inhibitors of metalloproteinase 1.
